# Supplementary material for: Heterospecific eavesdropping on an anti-parasitic referential alarm call
Source: Commun Biol. 2020 Mar 31;3:143. doi: 10.1038/s42003-020-0875-7 (PMC7109080; doi:10.1038/s42003-020-0875-7)
Supplement: Supplementary file 2 — Supplementary Information [file 42003_2020_875_MOESM2_ESM.pdf]

Supplementary Table 1. Pair-wise comparisons of non-zero latency data by treatments: Brown-headed cowbird chatters (BHCO), blue jay calls (BLJA), yellow warbler seets (SEET), red-winged blackbird chatters (RWBL), and wood thrush song (WOTH).

		z value	P value
Males	BLJA x BHCO	-0.64	0.967
	RWBL x BHCO	-1.26	0.706
	SEET x BHCO	-0.87	0.904
	WOTH x BHCO	5.12	< 0.001***
	RWBL x BLJA	-0.46	0.99
	SEET x BLJA	-0.10	1.00
	WOTH x BLJA	5.18	< 0.001***
	SEET x RWBL	0.38	0.995
	WOTH x RWBL	6.25	< 0.001***
	WOTH x SEET	5.59	< 0.001***
Females	BLJA x BHCO	0.63	0.970
	RWBL x BHCO	-1.91	0.305
	SEET x BHCO	0.14	1.00
	WOTH x BHCO	5.72	<0.001***
	RWBL x BLJA	-2.5	0.090
	SEET x BLJA	-0.47	0.990
	WOTH x BLJA	4.54	<0.001***
	SEET x RWBL	2.07	0.232
	WOTH x RWBL	7.45	<0.001***
	WOTH x SEET	5.32	<0.001***

Supplementary Table 2. Pair-wise comparisons of closest approach data by treatment

		z value	P value
Males	BLJA x BHCO	1.68	0.442
	RWBL x BHCO	-1.35	0.658
	SEET x BHCO	2.90	0.029*
	WOTH x BHCO	4.43	<0.001***
	RWBL x BLJA	-2.84	0.035*
	SEET x BLJA	1.01	0.846
	WOTH x BLJA	2.27	0.152
	SEET x RWBL	4.18	<0.001***
	WOTH x RWBL	5.81	<0.001***
	WOTH x SEET	1.25	0.718
Females	BLJA x BHCO	1.05	0.829
	RWBL x BHCO	-1.61	0.487
	SEET x BHCO	1.21	0.744
	WOTH x BHCO	2.10	0.219
	RWBL x BLJA	-2.56	0.076
	SEET x BLJA	0.14	0.999
	WOTH x BLJA	0.91	0.890
	SEET x RWBL	2.67	0.057
	WOTH x RWBL	3.57	0.003**
	WOTH x SEET	0.71	0.954

Supplementary Table 3. Pair-wise comparisons of calling rate by treatment

		z value	P value
2018 Males	RWBL x BHCO	4.37	<0.001***
	SEET x BHCO	-0.27	0.992
	WOTH x BHCO	-3.04	0.011*
	SEET x RWBL	-4.45	<0.001***
	WOTH x RWBL	-8.16	<0.001***
	WOTH x SEET	-2.66	0.037*
2019 Males	BLJA x BHCO	-1.74	0.405
	RWBL x BHCO	2.73	0.048*
	SEET x BHCO	-0.44	0.991
	WOTH x BHCO	-7.63	<0.001***
	RWBL x BLJA	4.35	<0.001***
	SEET x BLJA	1.13	0.786
	WOTH x BLJA	-5.67	<0.001***
	SEET x RWBL	-2.98	0.023*
	WOTH x RWBL	-9.76	<0.001***
	WOTH x SEET	-6.29	<0.001***
2019 Females	BLJA x BHCO	1.78	0.381
	RWBL x BHCO	4.78	< 0.001***
	SEET x BHCO	1.64	0.466
	WOTH x BHCO	-1.82	0.360
	RWBL x BLJA	2.79	0.041*
	SEET x BLJA	-0.12	0.999
	WOTH x BLJA	-3.53	0.003**
	SEET x RWBL	-2.86	0.033*
	WOTH x RWBL	-6.43	< 0.001***
	WOTH x SEET	-3.20	0.011*

Supplementary Table 4. Percentage of trials per playback treatment that non-focal species responded aggressively to

	Playbacks				
Non-focal species	Brown-headed cowbird chatter	Blue jay calls	Yellow warbler chips	Yellow warbler seets	Wood thrush songs
Male brown-headed cowbird	59.46%	2.94%	3.23%	5.88%	0.0%
Female brown-headed cowbird	54.05%	0.0%	3.23%	0.0%	0.0%
Gray catbird	16.22%	20.59%	9.68%	2.94%	2.86%
Indigo bunting	8.11%	11.76%	29.03%	5.88%	0.0%
Northern cardinal	8.11%	0.0%	0.0%	0.0%	0.0%
Willow flycatcher	8.11%	2.94%	3.23%	0.0%	0.0%
Song sparrow	8.11%	2.94%	3.23%	2.94%	0.0%
Common grackle	5.41%	8.82%	0.0%	0.0%	0.0%
American robin	2.70%	14.71%	3.23%	0.0%	0.0%
Orchard oriole	2.70%	2.94%	0.0%	0.0%	0.0%
Baltimore oriole	2.70%	5.88%	0.0%	0.0%	0.0%
Cedar waxwing	2.70%	5.88%	0.0%	0.0%	0.0%
Blue-gray gnatcatcher	2.70%	2.94%	0.0%	0.0%	0.0%
Warbling vireo	2.70%	2.94%	6.45%	0.0%	0.0%
Chipping sparrow	2.70%	0.0%	0.0%	0.0%	0.0%
Blue jay	0.0%	29.41%	0.0%	0.0%	0.0%
Common yellowthroat	0.0%	5.88%	12.90%	2.94%	0.0%
Field sparrow	0.0%	2.94%	3.23%	0.0%	0.0%
Eastern kingbird	0.0%	2.94%	0.0%	0.0%	0.0%
American goldfinch	0.0%	0.0%	6.45%	2.94%	0.0%
Tufted titmouse	0.0%	0.0%	3.23%	0.0%	0.0%
Least flycatcher	0.0%	2.94%	0.0%	0.0%	0.0%
Carolina wren	0.0%	2.94%	0.0%	0.0%	0.0%
Wood thrush	0.0%	0.0%	0.0%	0.0%	20.0%
